# The Book World of Medicine and Science

**Published:** 1893-06-10

**Authors:** 


### THE BOOK WORLD OF MEDICINE AND SCIENCE.

### BOOK REVIEWS.

HOBLYN'S DICTIONARY OF MEDICAL TERMS. By J. A. P. PRICE. 12th edition, revised, 1892, 10s. 6d. (Whittaker and Co.)

A definite and practical proof that this work has been properly appreciated by the medical world is to be found in the fact that it has been considered necessary to issue this, the twelfth edition. This issue, which we owe to the hand of Dr. Price, of Reading, differs but little from the editions which have preceded it. It has, however, been brought well up to date, and contains what will be found invaluable to many a tyro, namely, a full vocabulary of the words and phrases lately coined to meet the necessities of that infant branch of medical science, namely, bacteriology, the nomenclature involved in which, with its extensive technique, has been, and still is, a matter of no easy digestion nor assimilation. Invaluable, therefore, in its present form, is a medical dictionary of such size as to be no great incubus in the student's portable library, and published at such a price as to come within the easy range of all. Edited and re-edited as this work has been by classical scholars, the etomological and philological treatment of our medical parlance may be safely left in the hands of the compilers of this work, who, profiting by an Oxford education, may be trusted to exercise a vigilant and discretionary scrutiny over our medical phraseology, polluted as it has been from time immemorial by gross anachronisms and hybrid impurities of expression. We can confidently recommend this little volume to each and every student of medicine who takes an intelligent interest in the words he ought to use, and in the expressions which he is by necessity bound to hear.

### FIRST IMPRESSIONS OF BOOKS RECEIVED.

[It is requested that all books intended to be noticed in this column may be sent to the Editor, at the Office, 428, Strand, W.C., not later than Tuesday evening in each week.]

POPULAR INFORMATION CONCERNING INFECTIOUS DISEASES. By HORACE SWORDER, L.R.C.P., M.R.C.S., L.S.A., Medical Officer of Health to the Borough of Luton, &c. (Henry Renshaw, London, 1893.)

The title aptly expresses the object with which this little book is written. The arrest of infectious diseases is largely in the hands of the public, and "we must educate our masters." Mr. Sworder has brought together in a simple form, in language "understanded of the people," the chief facts about the common infectious diseases, and about methods of disinfection. The book is vouched for by Dr. Collingridge and dedicated to Dr. Broadbent, which is a significant testimony to its reliability and accuracy.

THE SANITARY INSPECTOR'S HANDBOOK. By ALBERT TAYLOR, Associate Sanitary Institute, Chief Sanitary Inspector to the Vestry of St. George, Hanover Square, London, &c. With illustrations. (London: H. K. Lewis, 1893.)

The sanitary inspector is abroad, and what more necessary than to provide him with a proper *vade mecum?* It is this

THE BOOK WORLD OF MEDICINE AND SCIENCE.—(*continued*).

that Mr. Taylor has attempted, and his large experience, both at Wigan and London, specially qualifies him for the task. The book deals with all the duties of a sanitary inspector from legal and sanitary standpoints. It is clearly written, and appears to be sufficiently full without being overloaded with details. It is essentially a practical book for practical men on one of the most practical subjects of the day.

### THE JUNE REVIEWS.

The Nineteenth Century is a strong number this month. Dr. Martineau's criticism of the newly found Gospel of St. Peter is an important contribution to the rapidly increasing number of comments called into being by these fragments. Mr. Henniker Heaton has a powerful denunciation of red-tapeism under the head of "Post Office Plundering and Blundering." Perhaps his strongest point is the urgent necessity for cheap postal facilities for small producers. It is startling to be reminded that we are paying £30,000,000 a year to the foreigner for dairy and garden produce which the British cultivator might supply if an Agricultural Parcels Post were in existence at a reasonable rate. Three other articles on the Rothampstead experiments, on wages, and on capital also discuss the agricultural question. Dr. Tuke, writing on habitual drunkards, strongly urges the necessity for further legislation on the ground that after fourteen years' experience, the Habitual Drunkards Act has produced only trivial and unsatisfactory results. He expresses the opinion that no measure will receive the assent of the Legislature which proposes that a person should be confined for what to the public mind verges on crime, on the mere application of friends or relatives, whatever the restrictions may be. "All such inquiries should be conducted in public, before a court presided over by a judge, assisted by two persons, medical or legal, one of whom should be appointed by the petitioner and one by the alleged drunkard. If this tribunal, after hearing evidence taken on oath, finds by a majority that the subject of inquiry is so far given over to habits of intemperance in stimulants or sedatives, as to render him unable to control himself, to make him dangerous to others, or to prevent him from managing his estate, it should be empowered to place him under restraint in such an establishment as should be determined by the court for a period not exceeding two years." Dr. Tuke considers that it is certain a small proportion can be cured, and that a compulsory measure would act as a powerful deterrent.

In The Century for this month Dr. Prudden calls attention to the need for a National Bureau of Health in the United States. Congress, it seems, has been much exercised in devising a system of national quarantine in view of the approaching cholera. It is urged that such work can only rightly be performed by a national Board supplementing and controlling the work of the local authorities. Although the due organization of quarantine is at present the most urgent requirement, the endowment of research and the establishment of a museum of hygiene and sanitary appliances are contemplated by Dr. Prudden as within the scope of the proposed Bureau. At present sanitation appears to a matter solely of local effort and responsibility in America. Dr. Prudden has a word of commendation for the English system. "Some of us admire, others wonder at the courage

THE BOOK WORLD MEDICINE OF AND SCIENCE—(*continued*).

and placidity with which England faces a threatened invasion of cholera. This is because she is ready to encounter it, not only with intelligent sanitation well under control all over the land, but because she meets it as a unit, and not as we are still forced to do, in haphazard fashion, as the resources and the sanitary intelligence of a single state decree, or as the whim of an autocratic officer may dictate."

